# Arrayed Force Sensors Made of Paper, Elastomer, and Hydrogel Particles

**DOI:** 10.3390/mi8120356

**Published:** 2017-12-08

**Authors:** Xiyue Zou, Tongfen Liang, Nastassja Lopez, Moustafa Ahmed, Akshitha Ajayan, Aaron D. Mazzeo

**Affiliations:** Department of Mechanical and Aerospace Engineering, Rutgers University, 98 Brett Road, Piscataway, NJ 08854, USA; xz289@scarletmail.rutgers.edu (X.Z.); tl393@scarletmail.rutgers.edu (T.L.); nl299@rutgers.edu (N.L.); mga9600@gmail.com (M.A.); akshitha.ajayan97@gmail.com (A.A.)

**Keywords:** electronic skin, paper-based electronics, force sensor, hydrogel, elastomer

## Abstract

This article presents a sensor for detecting the distribution of forces on a surface. The device with nine buttons consisted of an elastomer-based layer as a touch interface resting on a substrate of patterned metallized paper. The elastomer-based layer included a three-by-three array of deformable, hemispherical elements/reliefs, facing down toward an array of interdigitated capacitive sensing units on patterned metallized paper. Each hemispherical element is 20 mm in diameter and 8 mm in height. When a user applied pressure to the elastomer-based layer, the contact area between the hemispherical elements and the interdigitated capacitive sensing units increased with the deformation of the hemispherical elements. To enhance the sensitivity of the sensors, embedded particles of hydrogel in the elastomer-based layer increased the measured electrical responses. The measured capacitance increased because the effective dielectric permittivity of the hydrogel was greater than that of air. Electromechanical characterization verified that the hydrogel-filled elastomer was more sensitive to force at a low range of loads (23.4 pF/N) than elastomer alone without embedded hydrogel (3.4 pF/N), as the hydrogel reduced the effective elastic modulus of the composite material by a factor of seven. A simple demonstration suggests that the force-sensing array has the potential to contribute to wearable and soft robotic devices.

## 1. Introduction

This article describes the design, fabrication and experimental characterization of a force-sensing transducer made of metallized paper, elastomer and hydrogel for skin-like sensors. The device consisted of a metallized-paper-based substrate and an elastomer-based interface that included three-by-three deformable, hemispherical elements/reliefs. We filled the hemispherical elements with hydrogel particles to adjust the mechanical and electrical properties of the elastomer. When a user compressed the elastomeric layer, the contact area between the compressed elements/reliefs and the interdigitated capacitive sensing units etched on the metallized paper increased with the deformation of the hemispherical elements. The measured capacitance increased because the effective dielectric constant of the hydrogel (sodium polyacrylate) was larger than that of air. 

Skin, the largest organ of the human body, is capable of detecting human-environment interactions, but current humanoids, prosthetics and wearable devices lack spatial sensors with capabilities comparable to human skin. There are ongoing efforts to reduce cost of, complexity of and impediments to the manufacturability of skin-like sensors on substrates ranging from plastic films, to elastomers, to glass, to paper [1,2,3,4,5,6,7,8,9,10,11,12,13,14,15]. An ideal skin-like tactile sensor might be flexible, scalable, capable of detecting force/pressure and provide protection. Some skin-like tactile sensors make use of resistive/piezoresistive and capacitive effects to convert mechanical loading to electrical responses. Wang et al. fabricated paper-based piezoelectric touch pads with zinc oxide nanowires (the sensitivity was 0.57 nA/N) [16]. Pan et al. presented a resistive pressure sensor based on a micro-structured, conducting thin film of polymer with embedded hollow spheres (the sensitivity was 7.7–41.9 Ohm/kPa for applied pressures less than 100 Pa) [17]. Wang et al. reported an electronic skin that mapped applied pressure and provided a visual response through organic light-emitting diodes (the sensitivity was 42.7 Cd/m^−2^/kPa) [18]. D.-H. Kim et al. and J. Kim et al. reported classes of electronic systems that achieved thicknesses, effective elastic moduli, bending stiffnesses and areal mass densities matching epidermis [19,20]. Nassar et al. demonstrated a recyclable paper-based skin capable of detecting multiple environmental factors, such as temperature, humidity, acidity and pressure (the sensitivity was 0.61 pF/kPa for applied pressures in the range of 0–190 Pa) [21]. Kramer et al. and Y.-L. Park et al. developed hyperelastic pressure transducers that embedded silicone rubber with micro-channels of liquid-phase gallium-indium alloy [22,23]. The change in resistance was 0.07 Ohms when applying 40 kPa to the pressure sensor. Steckl et al. fabricated organic thin-film transistors on paper through dry-step processing [24]. Cheng et al. presented the development of a polymer-based capacitive sensing array that was capable of measuring normal and shear forces (the measured sensitivity was 1.67%/mN) [25].

Some of the previous approaches have high signal-to-noise fidelity in the transmitted measurements, but the devices required complex fabrication. The complexity stemmed from the need to embed flexible transistor- or oscillator-based circuits in strain-sensitive materials. As a result, these issues appear to limit the size of state-of-the-art synthetic skin (i.e., areas no larger than 300 mm by 300 mm). In other cases, the use of heavy metals (gallium and indium) is still more costly than sodium polyacrylate commonly used in baby diapers. In this work, the prototype was simple to fabricate and has potential as a low-cost, scalable, skin-like, pressure-sensing array for large-scale skin-like sensing.

Human skin detects external stimuli with mechanoreceptors and transmits the spatial intensity of pressure to the brain through neural networks [26,27]. Inspired by human skin, the device presented in this work is simple with the outer soft layer fabricated by molding/casting and the passive capacitive scanning circuits patterned on a paper-based substrate. The soft layer, or skin layer, like human epidermis and dermis, is a soft and smooth surface that covers the top of the device to provide a layer of cushioning. Underneath the skin layer, arrays of force-sensing units, similar to mechanoreceptors, convert the intensity of force/pressure to an electrical response [28]. The circuit layer, like a neural network underneath the dermis, transmits the signals from a force-sensing grid to a controller. In other words, the outer portion of the elastomeric layer of the device is like the epidermis that interfaces with the environment; the elastomer-metallized paper interface is like the dermis with internal mechanoreceptors; and traces in the metallized paper and associated circuitry are like the neural network underneath the dermis.

The paper-based capacitive sensing circuit is simple with the networks patterned into metallized paper and circumvents the complexity of active transistor-based circuits. Paper-based circuits, or papertronics, may also be suitable for mass production of disposable, flexible, biodegradable devices [29,30]. The fabricated devices were mechanically flexible and capable of detecting the distribution of force on a surface. The prototypes demonstrated a method of creating hybrid devices with molded/cast elastomers and paper-based substrates for future force-sensing, skin-like sensors.

## 2. Materials and Methods

**Figure 1:**
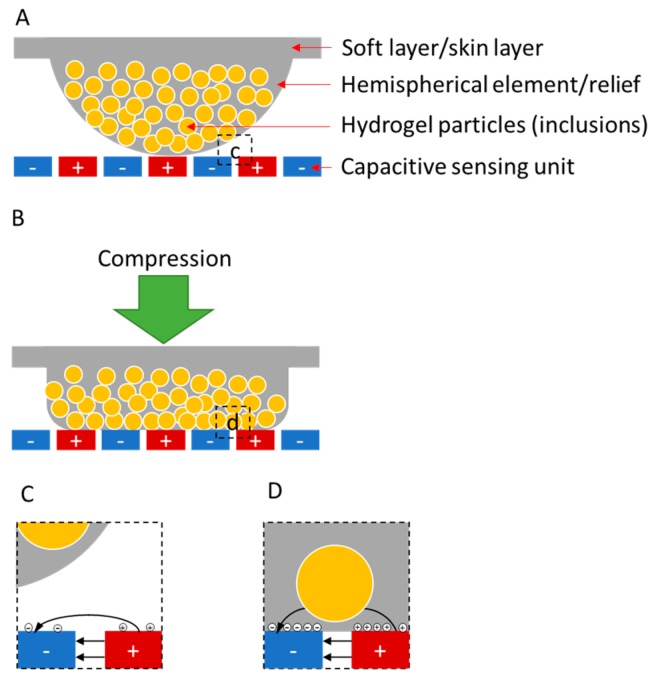
Profile of a force-sensing button. (**A**) Each sensor/button consists of a soft layer (skin-like interface) and a layer of metallized paper with patterned capacitive sensing units. The hemispherical element may or may not contain hydrogel particles (inclusions); (**B**) Compression of a button. When an element/relief compresses against the capacitive sensing units in the metallized paper, the element deforms to increase the area of interfacial contact; (**C**,**D**) Interaction between a capacitive sensing unit and a portion of a hemispherical element. As a hemispherical element/relief makes contact with a capacitive sensing unit, the stored electrical field/charge increases, which results in an increase in measured effective capacitance.

The device consisted of a metallized paper-based substrate and an elastomer-based structure that included three-by-three deformable, hemispherical elements/reliefs. The experimental design of the skin-like sensors in this work made use of three principles, as shown in Figure 1. First, the devices used passive capacitive sensing arrays with multiplexed measurements for detecting variations in electrical impedance over a two-dimensional area. Second, each hemispherical soft element/relief sat above an individual, interdigitated capacitive sensing unit to form a force-sensing button. When applying force to the backside of soft elements/reliefs, its contact area with the sensing unit increased with compression. As a result, the capacitance of the sensor increased because the effective dielectric constant of the soft material was higher than that of air. Third, by embedding inclusions of high dielectric constant into the soft material matrix, we increased the sensitivity of the sensors for detecting small forces. Hydrogel, a hydrophilic structure that is capable of holding a large amount of water in its network, was an ideal candidate for the inclusions because of its high dielectric permittivity, modest conductivity and low elastic modulus [31,32,33]. Hydrogel particles embedded within the hemispherical elements or protruding from the surfaces contributed to an increase in the measured effective capacitance of the sensors because hydrogel had a higher conductivity and dielectric permittivity relative to pure elastomer.

**Figure 2:**
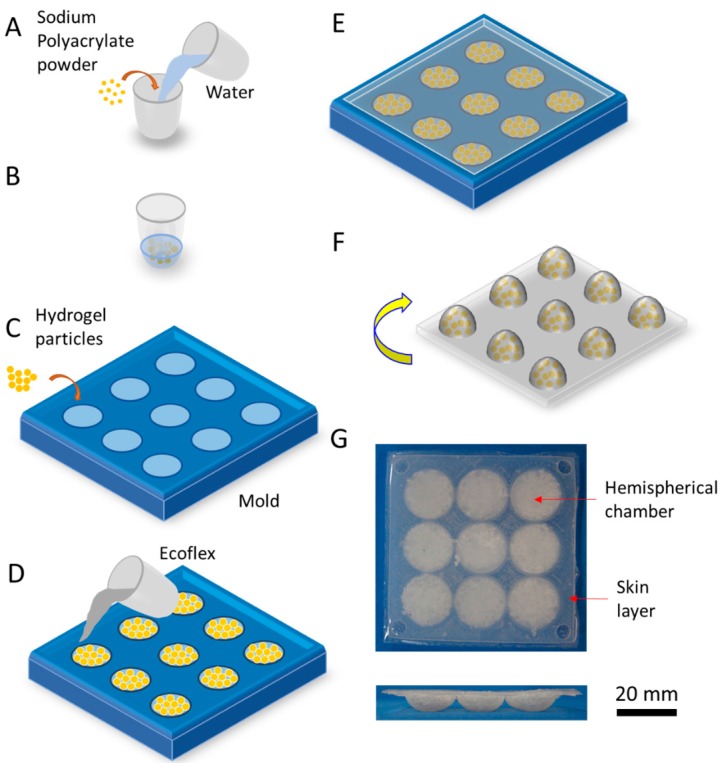
Fabrication of a soft layer using hydrogel-elastomer composites. (**A**) Combine powdered sodium polyacrylate with water; (**B**) wait 2 min for gel to form; (**C**) fill hemispherical chambers of the printed mold with hydrated hydrogel particles; (**D**) pour the prepared Ecoflex into the mold; (**E**) wait 30 min for Ecoflex to cure in an oven at 60 °C; (**F**) remove the soft composite material from the mold; (**G**) photograph of the soft layer in top view and side view.

To fabricate the composite material for the soft layers with hemispherical reliefs, we embedded hydrogel particles in Ecoflex^®^ 00-30 (Smooth-On, Inc., Macungie, PA, USA). As shown in Figure 2, we mixed 1 g of sodium polyacrylate (Universe of Science, Inc., Moravian Falls, NC, USA) with 10 g of water. After 2 min, the mixture grew into gel particles. The size of the particles ranged from 0.6 mm to 1.2 mm, as shown in Figure S1. We filled the hemispherical chambers of a 3D-printed mold (see Figure S2) with the same amount of gel particles and then filled and covered them with Ecoflex. Degassing the chambers with the material by a vacuum pump for 15 min eliminated the air voids inside the Ecoflex or Ecoflex-hydrogel composite. After the Ecoflex cured in an oven at 60 °C for 0.5 h, we separated the composite material from the mold. To avoid absorption of water from the hydrogel particles into the metallized paper, we let them sit for 24 h before testing. For the data shown in the main text of this article, the relative humidity in the room was approximately 55%. For repetitive data shown in Figure S3, the relative humidity was approximately 30%. 

To fabricate the paper-based capacitive sensors with interdigitated electrodes, we used laser-based ablation (Versa VLS 2.3 Laser Cutter, Universal Laser Systems, Inc., Scottsdale, AZ, USA) to etch through the conductive layer of metallized paper (AR Metallizing, Ltd., Franklin, MA, USA; see Figure S4). This technique for fabricating the paper-based capacitive sensing units is in a similar fashion as previous work [4,29]. The laser with the appropriate parameters (12.6% power, 100% speed and 500 PPI (pulses per inch)) cut through a top insulating coat and removed a 10 nm-thick layer of aluminum from the metallized paper without completely penetrating through the cellulose-based substrate. We brushed Silver Conductive Adhesive 503 (Electron Microscopy Sciences, Hatfield, PA, USA) on the surface of the conductive layer to attach wired leads.

**Figure 3:**
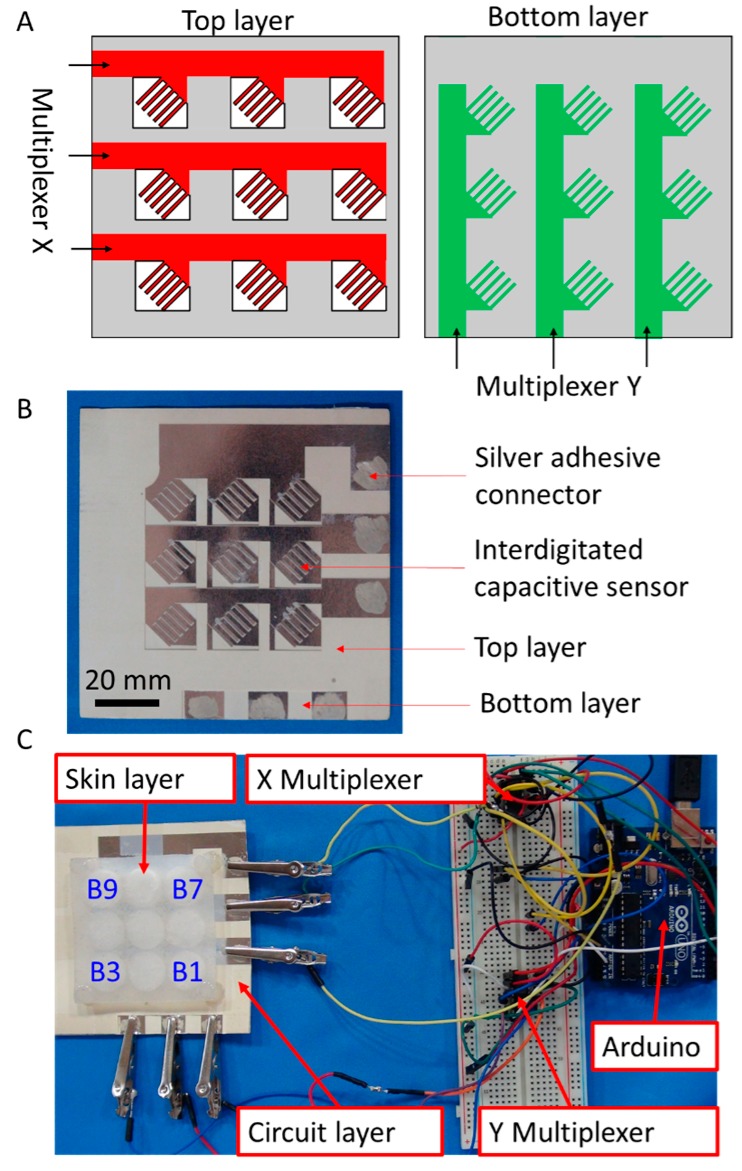
A diagram and photograph of a three-by-three arrayed sensor for measuring force. (**A**) Diagram of two multiplexers and stacked sheets of patterned metallized paper. The top and bottom layers connect with a multiplexer to reduce the number of required wires and demonstrate the scalability of the electromechanical sensors. The white areas in the top layer are the removed regions. The grey areas in both layers are the partially-ablated regions; (**B**) Photograph of the circuit; (**C**) Photograph of the measurement system.

As shown in Figure 3A, the capacitive scanning circuit interfaced with two layers of patterned metallized paper with the X layer stacked on top of the Y layer, thus making a three-by-three grid with each button having parallel, interdigitated metallized tines (see Figure S5). The sensitivity of the interdigitated capacitive sensors increased with the number of tines, but increasing the number of tines by decreasing their width made them more fragile. The design presented in this work used five tines to balance the sensitivity and the durability of the sensors. Each wire running to an X/Y layer tied all the buttons in a given column/row of the grid. All wires connected to each layer ran through an ADG608 Multiplexer (Analog Devices, Inc., Norwood, MA, USA). An Arduino Uno controlled these two multiplexers and measured the effective capacitance at each button. To measure the capacitance, an analog output from the Arduino sent a voltage impulse to one electrode of the capacitive sensor. An analog input measured the voltage of the other electrode. Figure 3B,C show the circuit and the measurement system, in which the soft layer of composite material was on the top of the circuit.

## 3. Results

To characterize the electromechanical relationship between compression and the measured capacitance of an individual sensor/button, we used an Instron 4411 (Instron, Norwood, MA, USA) material-testing machine to apply compression to the top of the skin layer and simultaneously measured the capacitance of an individual sensor with a Hewlett-Packard 4192A LF impedance analyzer (Hewlett-Packard Company, Palo Alto, CA, USA). The contact between the load cell and the skin layer was a circular piece of acrylic with a diameter of 20 mm, which matched the diameter of the hemispherical elements. We tested an Ecoflex-based hemispherical element without embedded hydrogel and a composite hemispherical element of Ecoflex and hydrogel. The maximum compressive displacement in each test was 2.4 mm with a speed of 20 µm/s, and we repeated each test five times.

**Figure 4:**
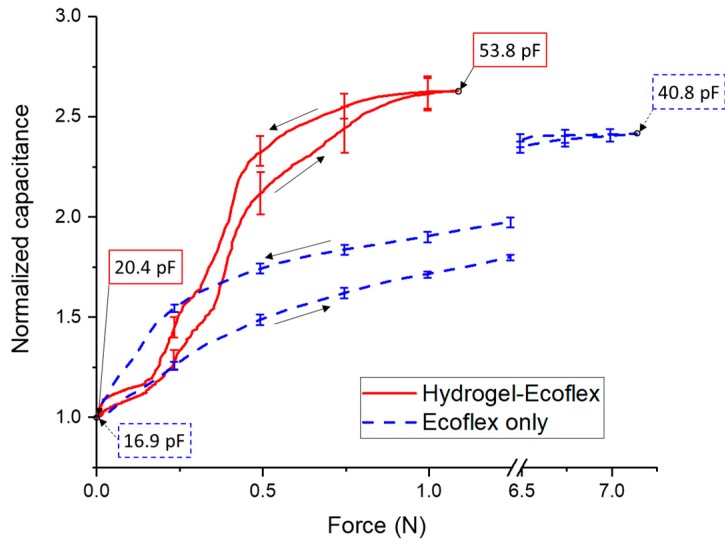
Electromechanical characterization of a single sensor/button. The tested button is in the middle/center of the three-by-three grid of the soft layer. The maximum displacement applied in each test was 2.4 mm. The upward arrow shows the compression cycle, and the downward arrow shows the release cycle. To make a meaningful comparison between the two cases (Ecoflex and hydrogel-Ecoflex hemispheres), there is a break in the x-axis from 1.25 N to 6.25 N. The plotted results are the average values of five repeated tests, and the error bars are ±1 standard deviations shown every 0.25 N. The normalized capacitance is the ratio of the measured value to the initial value. The frequency of excitation on the impedance analyzer was 100 kHz.

Figure 4 shows the electromechanical characterization of a button made from Ecoflex and a button made of an Ecoflex-hydrogel composite. The latter had a higher rate of increasing capacitance than the former. For the composite button, the contact area between hydrogel particles attached to the hemispherical elements and the electrodes of the sensor gradually increased with pressure, so the capacitance increased, as well. Compared to the element made of pure Ecoflex, the capacitance-to-force curves of the composites are not smooth because of the uneven distribution of the hydrogel particles embedded within the Ecoflex. To depict the capacitive response of a sensor, we use normalized capacitance, which is the ratio of the measured capacitance to the initial capacitance. As shown in the figure, the sensitivity of the composite material at low forces greater than ~0.2 N was higher than that of Ecoflex 30 by itself. The sensitivity of the hydrogel-Ecoflex force sensor also gradually decreased when the force was larger than 0.5 N and where the displacement of the loading cell was 1.85 mm. When fully compressed at 2.4 mm, the results also suggest that the hydrogel particles provided a modest enhancement of the electric field between the interdigitated electrodes of the material, as the normalized capacitance (~2.6) with the composite buttons was higher than that (~2.4) of the button with only Ecoflex. 

**Figure 5:**
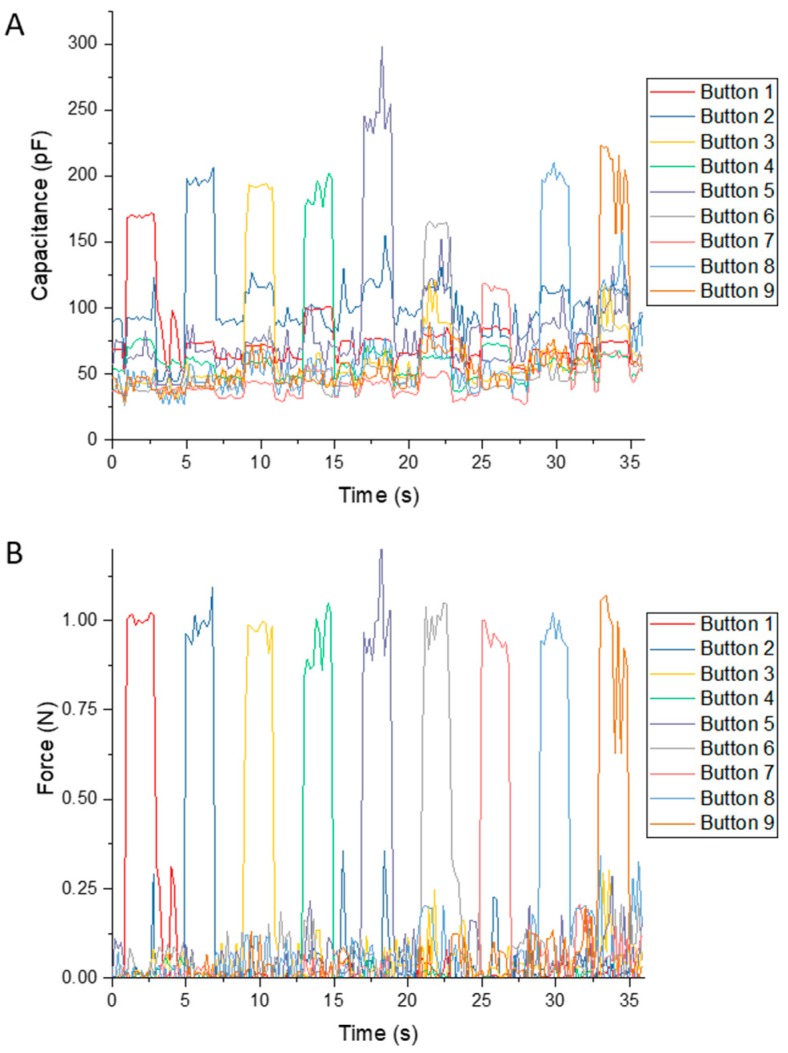
The response of the force-sensing array and its calibrated results. (**A**) The response of the force-sensing array to a load of 1 N placed on individualized buttons/sensors; (**B**) the calibrated results of the force sensing-array rebuilt by Tikhonov regularization.

To characterize the response of a prototypical sensing array, we used a capacitive scanning circuit to monitor the responses at all the buttons, while a single load acted on individual buttons. We applied a weight of 0.98 N (100 g) to the top of each sensor and measured the normalized capacitances on all nine channels. As shown in Figure 5A, there were varied responses to the loads applied on distinct buttons, but in every case, the greatest measured change in normalized capacitance was on the channel associated with the button receiving the mechanical load. To demonstrate the stability of the devices, we show the normalized capacitances of a single button in repetitive testing (see Figure S3). The variation in the sensitivity stemmed from the uneven distribution of hydrogel particles in each hemispherical element. Additionally, when applying pressure on one button, other buttons also exhibited slight responses because of the continuous nature of the elastomeric interface. To rebuild a uniform and correct signal, it was necessary to calibrate the sensitivity of each sensor.

To calibrate the sensitivity of each sensor, we used regularization to convert the measured capacitance to applied force. In the forward problem, we applied the same weight to each sensing point and recorded the capacitances to establish elements in each row of a nine-by-nine sensitivity conversion matrix *S*. The problem is equivalent to solving the equation:

(1) c=S×f

where *c* is a column vector ${\left[\begin{array}{ccc} c_{1} & c_{2} & \begin{array}{cc} \cdots & c_{n} \end{array} \end{array}\right]}^{T}$ for the measured capacitance minus the initial capacitance of each sensor, *S* is an *n*-by-*n* matrix that converts force to capacitance at each sensing point and *f* is a column vector ${\left[\begin{array}{ccc} f_{1} & f_{2} & \begin{array}{cc} \cdots & f_{n} \end{array} \end{array}\right]}^{T}$ in which each element is the force applied to each button. Each element in vector *c* follows expression $c_{i}=C_{i}-C_{i}^{Z}$, where $C_{i}$ is the measured capacitance of button *i* and $C_{i}^{Z}$ is the initial capacitance of button *i*. In the matrix *S*, the number in the *i*th row and the *j*th column means the capacitance of button *j* when applying force to button *i*. In this framework, we also normalized forces and capacitances in the calculation. 

In the inverse problem, we derived the distributions of forces from the measured capacitances and the matrix *S*. To solve the inverse problem, we used the Tikhonov regularization algorithm, which follows from this equation [34,35]:

(2) $$\hat{f}={\left(S^{T}S+\lambda I^{T}I\right)}^{-1}S^{T}c$$

where $\hat{f}$ is the estimation of the force distribution and λ is Tikhonov regularization factor. The factor we chose was 0.002 to balance the accuracy and noise ratio. Figure 5B shows the force signals derived from the measured capacitances. The regularization converts the uneven sensitivity of different buttons to the uniform outputs of force.

**Figure 6:**
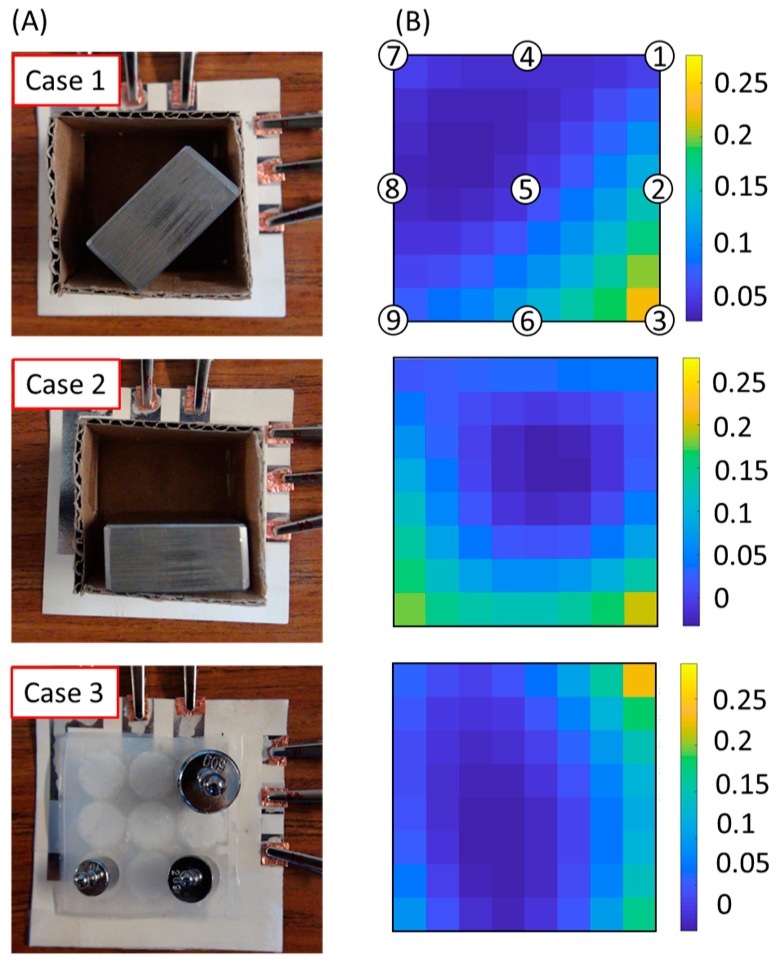
Demonstration of a force-sensing array. (**A**) For the first two cases, a metal bar was in two different positions in a cardboard box. The mass of the load was 284 g, and its dimensions were 19 mm × 37 mm × 52 mm. For the third case, the weights were 10 g, 20 g and 50 g. (**B**) The calculated distribution of forces retrieved from the capacitive measurements and regularization. The color maps from the top downwards show the distribution of normalized force on the sensor array corresponding to Case 1, Case 2, and Case 3, respectively. We used interpolation to fill the gaps between buttons. The buttons were in the center and along the edge of the color maps. The color bar is in terms of normalized force.

A simple demonstration shows that the prototyped sensing array was capable of measuring a distribution of forces. As shown in Figure 6, we placed a cardboard box on the top of the skin layer. We then put a metal bar at different locations within the box to manipulate the distribution of forces acting on the buttons. With the regularization method, we retrieved and calculated the distribution of forces using the measured capacitance vector. Case 1 and Case 2 show simple applications of the device that can detect the distribution of weights in a cardboard box. The stiffness of the box is much higher than that of the elastomer, so the distribution of forces on the elastomer is different from that when placing discrete weights on the elastomer without the box. We can see some high-force points at un-loaded points because the box applied compression at these locations. Case 3 demonstrates how the device was able to detect distributed pressures on the elastomer. Video S1 demonstrates the functionality of the force-sensing array being pushed by fingers.

## 4. Discussion

The process of fabrication described in this work is capable of altering the electromechanical relationship between applied forces and measured changes in capacitance. The sensitivity to applied force was dependent on changes in the material properties of the hydrogel-elastomer composite. As shown in Figure 4, to cause the same displacement of 2.4mm, the force (~1.3 N) associated with the hydrogel-elastomer composite was much less than the 7 N in the case of pure elastomer. This observation suggests that the effective elastic modulus or stiffness of the hydrogel-elastomer composite was much less than that of pure elastomer. Thus, one mechanism for the increased sensitivity to force at low loadings is that the softer hydrogel-elastomer composite deformed more at low loads than the pure elastomer, which caused the initially hemispherical surface to make significant interfacial contact with the capacitance-sensing units. Furthermore, sensitivity to displacement was not as large as sensitivity to force, as the normalized capacitances at the same maximum displacements of 2.4 mm did not differ nearly as much as the ratio of the maximum applied forces required to get to these maximum displacements for the case with hydrogel particles and the case without hydrogel particles. 

Another hypothesized mechanism for increased sensitivity to force was the change in electrical properties in the soft layer introduced with the inclusion of hydrogel-based particles. As the permittivity of water is greater than that of air by a factor approximately of 80 and salty water is significantly more electrically conductive than air, we expected significantly higher values of measured capacitance in experiments with the hydrogel-elastomer composite. While we did observe higher absolute values of capacitance at the start and maximum displacement in the hydrogel-elastomer composites than in the pure elastomer as shown in Figure 4, these measured capacitances did not reflect the expected innate differences in electrical properties between elastomer and hydrogel. The likely cause for the limited increases in measured capacitances was the relative positions between the capacitance-sensing units and the soft dielectric/conductive media. The metallized paper still had a polymer-based top layer over the evaporated aluminum, and there was likely still elastomer, polymer coating or air in the paths of the dominant electric fields between interdigitated electrodes.

## 5. Conclusions

In this article, we have introduced a skin-like, force-sensing array that is flexible, low-cost, easy-to-fabricate and capable of measuring distributions of forces over a two-dimensional area. The device consisted of an elastomeric soft shell embedded with hydrogel particles and a metallized-paper-based capacitive scanning array. The inclusion of hydrogels increased the sensitivity of the force sensor at low loads with the inclusion of hydrogel particles. The hydrogel reduced the effective elastic modulus of the composite material and increased the effective permittivity of the elastomer-hydrogel composite. The uneven sensitivity of the capacitive sensors stemmed from the non-uniform distribution of hydrogel particles attached to the hemispherical elements. To improve the uniformity of the sensitivity, a possible solution for a future study might use a molded hydrogel in a hemispherical shape instead of embedding hydrogel particles in elastomer. Another potential issue is that the sensitivity of the device may change with humidity, as hydrogel particles dehydrate when the humidity decreases. As a result, the process of calibration is necessary before testing. In the future, skin-like sensors made of paper, elastomer and hydrogel particles may aid in measurements of distributive forces. These sensors have the potential to enhance human-machine interactions (e.g., prosthetics, robots, haptics) or detect damage to structures or vehicles for structural health monitoring of soft material-based systems. 

## Supplementary Materials

The following are available online at www.mdpi.com/2072-666X/8/12/356/s1, Figure S1: Microscopic images of hydrogel particles, Figure S2: Photograph of a 3D-printed mold with polylactic acid (PLA), Figure S3: The stability of the devices, Figure S4: Diagram depicting the cross-sectional morphology of metallized paper, Figure S5: Photograph of paper-based circuits patterned into metallized paper, Video S1: Demonstration of a paper-based force-sensing array.
