# Supplementary material for: Arrayed Force Sensors Made of Paper, Elastomer, and Hydrogel Particles
Source: Micromachines (Basel). 2017 Dec 8;8(12):356. doi: 10.3390/mi8120356 (PMC6187906; doi:10.3390/mi8120356)
Supplement: Supplementary file 1 [file micromachines-08-00356-s001.zip › micromachines-227500-supplementary/micromachines-227500 supplementary materials.pdf]

micromachines

Arrayed Force Sensors Made of Paper, Elastomer, and Hydrogel Particles

Xiyue Zou, Tongfen Liang, Nastassja Lopez, Moustafa Ahmed, Akshitha Ajayan and Aaron D. Mazzeo

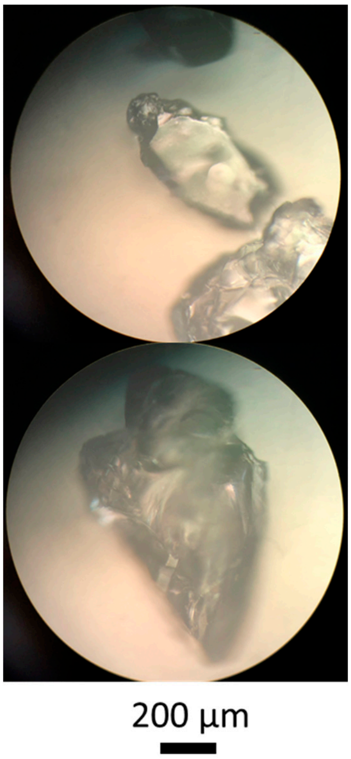

Figure S1. Microscopic images of hydrogel particles. The size of the hydrogel particles ranged from 0.6 mm to 1.2 mm.

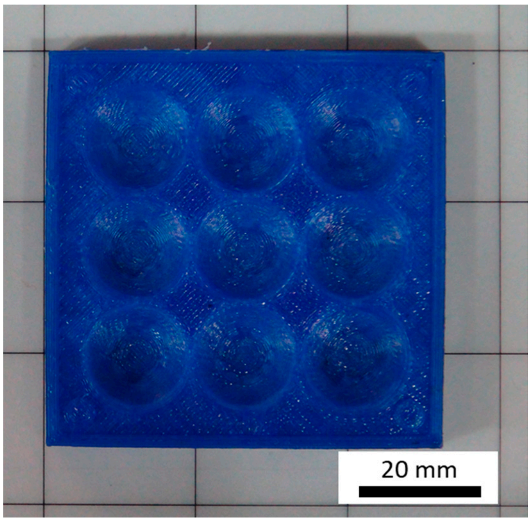

Figure S2. Photograph of a 3D-printed mold with polylactic acid (PLA).

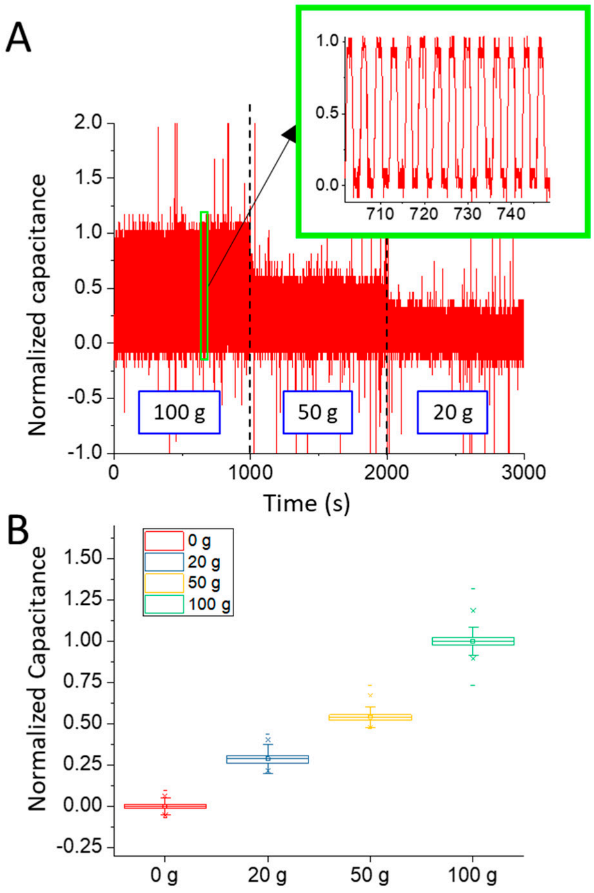

Figure S3. The stability of the devices. The repetitive tests demonstrate the stability of the sensor. After hydrating the buttons, the device sat for 24 h in a room with relative humidity of approximately 30%. During the test, weights sat on top of a button for 2 s, and weights came off the button for 2 s. (**A**) The normalized capacitance of a button over 3000 s. The applied weights came from masses of 100 g, 50 g, and 20 g. (**B**) A box chart of normalized capacitances associated with different masses/applied weights. Although the values for normalized capacitance in this plot appear to be stable, their absolute values were significantly different from those shown in Figure 5. This difference may be a result of the aging elastomer and hydrogel particles, along with a low relative humidity in the surrounding environment.

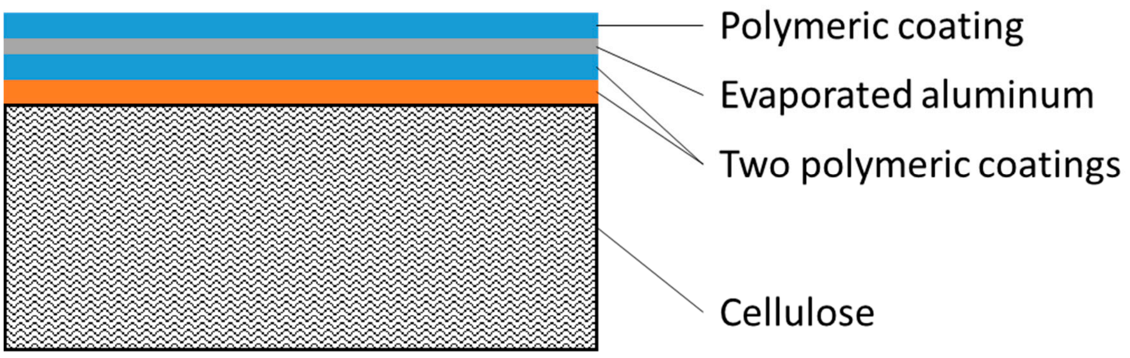

Figure S4. Diagram depicting the cross-sectional morphology of metallized paper. The metallized paper consisted of five layers, including cellulose, two polymeric coatings on the cellulose, evaporated aluminum, and one polymeric coating on the aluminum. The thickness of evaporated aluminum was approximately 10 nm. In this study, the thickness of the metallized paper was 150 µm. For more information, see [1,2].

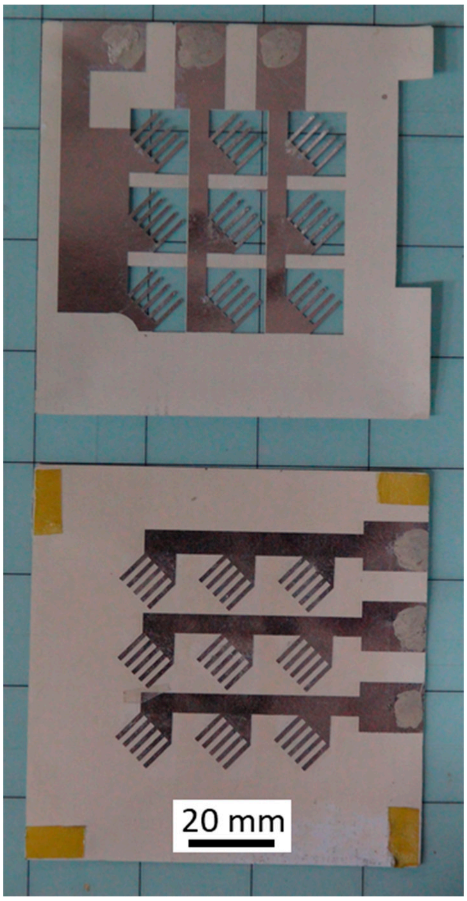

Figure S5. Photograph of paper-based circuits patterned into metallized paper.

References

1. Xie, J.; Chen, Q.; Suresh, P.; Roy, S.; White, J.F.; Mazzeo, A.D. Paper-based plasma sanitizers. *Proc. Natl. Acad. Sci. USA* **2017**, *114*, 5119–5124, doi:10.1073/pnas.1621203114.
2. Mazzeo, A.D.; Kalb, W.B.; Chan, L.; Killian, M.G.; Bloch, J.-F.; Mazzeo, B.A.; Whitesides, G.M. Paper-based, capacitive touch pads. *Adv. Mater.* **2012**, *24*, 2850–2856, doi:10.1002/adma.201200137.
